# Experimental and Numerical Study of Adhesively and Bolted Connections of Pultruded GFRP I-Shape Profiles

**DOI:** 10.3390/polym14050894

**Published:** 2022-02-24

**Authors:** Amir Reza Eskenati, Amir Mahboob, Ernest Bernat-Maso, Lluís Gil

**Affiliations:** 1 Strength of Materials and Structural Engineering Department, Polytechnic University of Catalonia, C/Colom 11, TR45, 08222 Terrassa, Spain; amir.mahboob@upc.edu (A.M.); ernest.bernat@upc.edu (E.B.-M.); lluis.gil@upc.edu (L.G.); 2 Serra Húnter Fellow, 08222 Terrassa, Spain

**Keywords:** pultruded FRP, bolt connection, adhesively connection, FE analysis, glass fiber

## Abstract

Recent developments indicate that the application of pultruded FRP profiles has been continuously growing in the construction industry. Generating more complex structures composed of pultruded FRP profiles requires joining them. In particular, I-shape glass fiber pultruded profiles are commonly used and the possible joints to connect them should be specifically studied. The mechanical behavior of adhesively and bolted joints for pultruded Glass FRP (GFRP) profiles has been experimentally addressed and numerically modeled. A total of nine specimens with different configurations (bolted joints, adhesive joints, web joints, web and flange joints, and two different angles between profiles) were fabricated and tested, extending the available published information. The novelty of the research is in the direct comparison of joint technologies (bolted vs. adhesive), joint configuration (web vs. flange + web) and angles between profiles in a comprehensive way. Plates for flange joints were fabricated with carbon fiber FRP. Experimental results indicate that adding the bolted flange connection allowed for a slight increase of the load bearing capacity (up to 15%) but a significant increase in the stiffness (between 2 and 7 times). Hence, it is concluded that using carbon FRP bolted flange connection should be considered when increasing the joint stiffness is sought. Adhesively connections only reached 25% of the expected shear strength according to the adhesive producer if comparing the numerically calculated shear strength at the failure time with the shear strength capacity of the adhesive. Apart from assessing adhesive connections, the implemented 3D numerical model was aimed at providing a simplified effective tool to effectively design bolted joints. Although the accurate fitting between experimental and numerical results of the mechanical response, especially the stiffness of the joint, the local failure experimentally observed was not automatically represented by the model, because of the simplified definition of the materials oriented to make the model available for a wide range of practitioners.

## 1. Introduction

The first use of fiber reinforced polymer dated back thirty years ago, and nowadays, FRP material is employed by many engineers, technician and architects in order to strengthen and reinforce concrete structures [1,2,3,4,5]. Recently, there is a great interest on the applications of FRP profiles to produce hybrid structural systems. FRP-concrete structures improve the benefits of materials by combining FRP, which is highly tensile resistant and lightweight with low-cost compressive-resistant concrete [6].

Among FRP production technologies, pultrusion [7] is a consecutive process aimed to produce FRP longitudinal elements with constant cross-sections, by means of using continuous roving soaked with resin ad heated to cure them. Regarding the type of pultruded FRP elements, Alhawamdeh et al. [8] reported the I-shape profiles were the most used ones. Pultruded FRPs have several advantages apart from the general ones directly associated with FRP materials (including corrosion resistance, high strength to weight ratio and durability). Marra [9] proved that pultruded FRP decreased the structure mass by about 70% and increased the strength and stiffness. Keller et al. [10] reported that pultruded FRP materials that were used in bridge and building constructions remained effective up to 17 years after installation. Only small cracks were seen in some elements of the studied bridge.

Karimipour and Farhangi used a noble grooving methods such as EBR and EBROG on improving the performance of structures using GFRP materials [11]. Soraghi et al. [12] analyzied the bond response between the concrete and rebar against corrosion. Farhangi and Karakouzian reviewed the applications of the recycled material and GFRP on improving the structural resistance of structures [13].

However, other studies on I-shape pultruded FRP profiles noticed that the production technique also has its cons. Because of the roving-rich core at the heart of the web-to-flange junction, shear failure of this area is likely according with the experimental results reported by Turvey et al. [14] and Fascetti et al. [15]. In the same line, Alhawamdeh et al. [8] focused on the local buckling failure of I-section pultruded profiles identifying the web-to-flange area as the most critical one. In order to limit this problematic, Górecki et al. [16] proposed a sinusoidal-shaped web definition, which proved to be really effective.

One of the main applications so pultruded FRP profiles is concrete-FRP hybrid structures. In this line, Koaik et al. [17] did experimental tests on concrete-GFRP hybrid beams which were connected with epoxy adhesive and bolted elements. They concluded that the bonding and bolting mixed connection had better performance under flexural load. In addition, this mixed connection led to avoiding shear failure at the concrete-GFRP interface. Alachek et al. [18] performed experimental and finite element analyses to examine the effect of adhesive joints between pultruded GFRP and concrete beams under push-out shear test. Correia et al. [19,20] used GFRP I-profiles connected to concrete beams under three and four point flexural tests. They observed a considerable increase in stiffness and strength. Also, Correia [21] and Nunes [22] investigated the structural behavior of a bare GFRP beam and an hybrid carbon-glass FRP beam using unidirectional carbon fiber mats. They used experimental and numerical methods resulting in similar responses in terms of stiffness and ultimate load. Qin et al. [23] modeled hybrid FRP reinforced concrete beams to determine the effect of reinforcement ratio on the flexural performance. The over-reinforced design of the FRPRC hybrid beams was indicated as a preferred solution with high stability, high load volume and strong ductility behavior. Mahboob et al. [24] tested some CFRP-concrete hybrid slabs under three-point bending tests highlighting the possibility of using continuous flexible open fibers meshes to perform the concrete-FRP connection.

Hence, it is also necessary to connect FRP elements so as to give continuity to these strengthening applications or hybrid structural systems. Roca et al. [25] presented an in-depth review of the composite joint technologies being of special interest the relationship between the joint typology and the possible failure modes. In the same line, Sallam [26] presented an extensive review about composite joints, including bolted, bonded and hybrid possibilities. Apart from the definition of the different joints and the corresponding failure modes, Sallam also included a complete review of the calculation equations developed for different types of composite joints. Because of the initially observed failure modes of composite joints, several authors worked in the line of improving them. A comprehensive review of bolted composite joints that included practical production improvements was presented by Galinska [27].

Lee et al. [28] investigated the mechanical behavior of a pultruded fiber reinforced polymer (PFRP) single bolted connection under tension load. They concluded that the hole clearance had no significant effect. The same test setup was used for thermoplastic composites by Tobalina et al. [29] although their test design was oriented to a specific application. Bank [30] discussed FRP profile connections and considered some parameters were particularly influential to define limit states, including stress, load and resistance factor.

Moving from FRP to the particular case of GFRP, several experimental, analytical and numerical studies [31,32], were conducted on GFRP connections and the results showed that some parameters were especially relevant: fiber orientation, geometric parameters, hole clearance, washer size and connection angle were prove to be effective parameters to increase the strength of GFRP connections.

Apart from the bending and tensile mechanical characterization, other authors dealt with more complex phenomena, like fatigue. Zhang et al. [33] studied the fatigue response of adhesively-bonded pultruded connections subjected to different environmental conditions including temperature and moisture. Similarly, Wingerde et al. [34] investigated the fatigue behavior of pultruded FRP profiles joined with bolt connections and resin injection.

Other researchers tried to move furthest from the study of connections to the analysis of structural systems that incorporate such connections between FRP members. This was the case of the work presented by Martins et al. [35] who investigated the effectiveness of GFRP profiles when used on beam-to-column systems through experimental and numerical methods. This connection was also compared with a tailor-made steel connection showing that there were some factors which were effective to increase the strength of the connection or its rigidity, including the bolt edge distance. Mottram and Zheng [36] tested three full-sized beam-to-column connections including pultruded FRPs with steel flange cleats as a first approach to FRP connection technology. In another study, Qureshi and Mottram [37] demonstrated a significant increase in rotational stiffness and moment capacity on joints with FRP members.

Badifu et al. [38] investigated the failure mechanism of a pultruded FRP framework where joints were specifically analysed. Experimental results showed that the frame had an initial linear elastic behavior under vertical load, so the joints behaved elastically. Sousa et al. [39] studied the application of adhesively bonded connections using pultruded GFRP profiles for building systems, including experimental and analytical procedures showing that adhesive joints were a technically competitive option. Zhang et al. [40] studied three types of connection for beam-to-column using pultruded GFRP with epoxy resin and bolt connection and steel endplate. They compared experimental results with numerical analysis and concluded that thinner connection endplate resulted in a more ductile response. Hizam et al. [41] fabricated composite trusses with pultruded GFRP under two load cases in order to investigate the flexural bending capacity of this structure characterized by hinged-like composite joints. Additionally, they presented an analytical study that showed good agreement with the experimental results.

Finally, there are also researches completely focused on the numerical simulation of composite joints. This is the case of Feo et al. [42], who investigated the shear behavior of bolted composites using numerical methods to evaluate the distribution of shear stress between the bolts; the number of rows and the number of bolts were variable. The numerical results showed the load was not uniformly divided in multi-bolt joints. Moreover, the pressure washers had positive effect on stress distribution. In the simulation line, Li et al. [43] proposed an interesting simplified bidimensional model oriented to study the shear stress distribution for composite bolted joints. Moving to time-dependent response, Yu et al. [44] investigated the response of composite single-bolted joints under dynamic loads using Abaqus/Explicit software to take into account the damage progress around holes.

### Research Significance

After reviewing the state of the art, it was decided about the scope of the research. Because the most common production technique of FRP profiles is pultrusion and the most common material used is glass fiber reinforced polymer, GFRP pultruded profiles were chosen. In addition, the most common shape of the pultruded GFRP profiles is I-shape. Thus, pultruded I-shape GFRP profiles were selected to perform the current joint study. In addition, although there are a few reviews which combine information about studies on bolted and adhesive connections, there is little available literature that performed a direct comparison between adhesive and bolted joint types for composite connections. Thus, comparing these two types of joints for analogous connections is required. In addition, considering the available publications about joint design it was also believed that extending the direct comparable information between only-web connection and flange and web connections was required, so this issue was also planned as an aim of the research. Finally, there are some parameters like the angle between connected profiles, which is little researched, being aligned or perpendicular configurations the most common ones. Knowing the influence of the connection angle is a must for general development of joint technology, so it was another research topic included in the current work. Thus, an experimental campaign including GFRP connections with different geometries (angles), different technologies (bolted and adhesively) and different connection systems (at web or web and flanges) has been developed to contribute to the previously described knowledge gap. In addition, finite element method was used to model the experimentally observed response in order to provide a useful tool for practitioners far from current complex and specific simulation tools reported in the literature. Thus, the purpose of the implemented FE model is providing a simplified tool that is capable of reproducing complex experimental tests in order to perform numerical simulations instead of experimental campaigns in the future studies of composite joints.

## 2. Materials and Methods

### 2.1. Specimen Description

The experimental study aimed to investigate the structural behavior of bolted and adhesively bonded connections between glass fiber reinforced polymer (GFRP) pultruded I-profiles. A total of nine specimens were fabricated and tested. To label experimental specimens, the format ‘abcd’ is defined, where ‘a’ shows the angle between the two parts of the GFRP profile: 120° or 160° defined as the extreme values of the suitability range to produce arched structures for tunneling sustainment, which was the final application aim of a larger research program. This particular connection study belongs to, ‘b’ indicates where the connection was placed: web-W or web&flange-WF, ‘c’ defines the type of connection: bolted-B connection or adhesively-A connection. It was expected that bolted connections had more deformability but better durability in high moisture environments compared to adhesive connection, so assessing the stiffness of bolted vs. adhesive connections was required. Bolted connection may have initial settlement movements due to small gaps between holes and bolts. In contrast, adhesive connections have no initial gaps, which limits total deformation due to joint settlements, being an interesting alternative to consider. However, epoxy resins are not suitable to cure in high moisture environment, and it is also suspected that continuous water exposure may damage this type of resins [45], which would be a hazard for civil engineering applications. Hence, exploring bolted connections was also required and the comparison among these two options was of interest.

**Figure 1:**
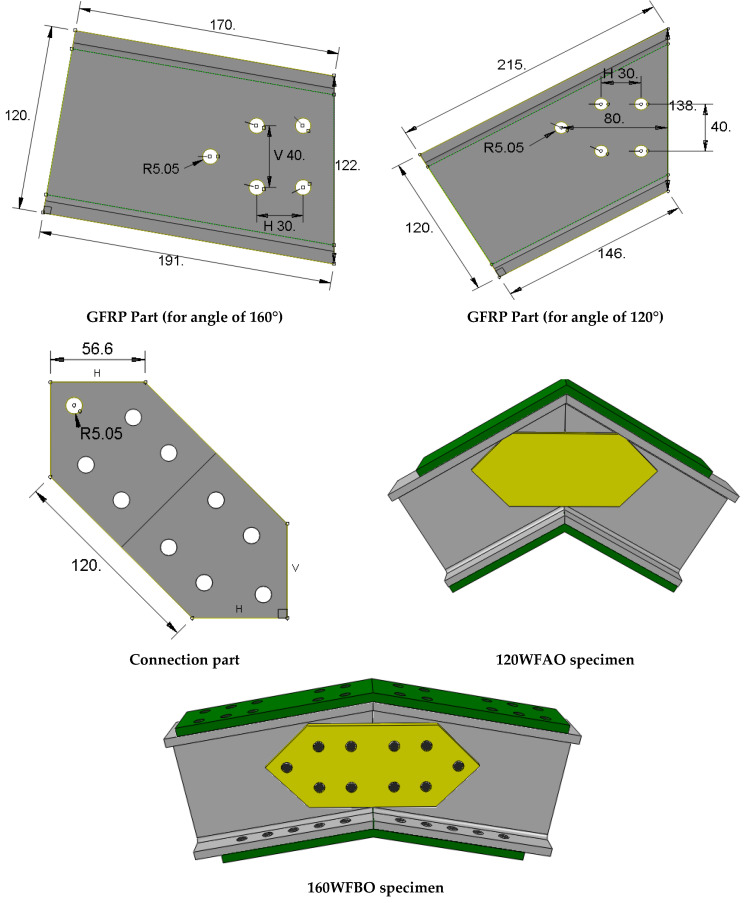
The geometry of the specimens (dimensions in mm).

Finally, ‘d’ shows the direction the load was applied: as per open-O the angle of the joint or to close-C it. One specimen 120WFBO was duplicated (120WFBO-2) in order to check the repeatability of the production and testing procedures. Figure 1 presents the specimens details. Table 1 shows all the details of the geometry of the parts used to mount the specimens.

### 2.2. Materials

#### 2.2.1. GFRP Pultruded Profiles

I-shape GFRP pultruded profiles were used to fabricate experimental specimens. GFRP pultruded profiles were composed of E-glass non-continuous fibers embedded into an isophthalic polyester resin matrix. Table 2 shows the main mechanical properties of GFRP pultruded profiles, which were previously obtained by Neagoe’s study [46]. Web connection pieces were cut from the web of the same GFRP profiles.

#### 2.2.2. CFRP Flange Connectors

To assure a complete geometric adaptation, flange connectors were produced ad-hoc for each specific pair of GFRP profile pieces. These were handmade and produced by a wet-lay-up lamination procedure. Previously, web connected profiles of the corresponding specimens were used as counter-mold with demolding agents applied. Alternate layers of unidirectional carbon fiber MasterBrace FIB 300/50 CFS [47] and brushed epoxy resin MasterBrace P3500 [48] were applied in a weight ratio of 50% to 50% to ensure complete resin penetration even in handcrafted production. Layers of fiber were placed along the longitudinal direction of the profiles, resulting in a 6 mm thick laminate. The produced CFRP angular laminate was cured under indoor environmental conditions for 1 week before perforating holes and unmolding. Tensile tests (EN ISO 527-1:2012) on 5 rectangular plates obtained from produced laminates resulted in an average tensile strength of 1120 MPa and an elastic modulus of 45.55 GPa. In this research CFRP was used as flange connector because it shows more stiffness than other FRPs and it was aimed to study if this may affect the stiffness of the jont. The main properties of carbon fiber and epoxy resin used to produce flange connectors are summarized in Table 3 and Table 4 respectively.

#### 2.2.3. Screws, Nuts and Washers

Among connection methods, bolted connections are commonly used because of their advantages, including easy installation and high strength. Tensile and shear loads should be considered in the design of bolted connections [49]. The bolted connection consisted of M10 × 40 and M10 × 35 hexagonal head screws with quality Q12.9. Corresponding M10 nuts and M10 washers were also used to mount the bolted joints between the GFRP pultruded profiles.

#### 2.2.4. Adhesive Epoxy Resin

The bicomponent epoxy resin MasterBrace ADH 4000 was used as the adhesive to bond the web and flange connectors of the studied specimens with adhesively joints. The main properties of this resin are summarized in Table 5.

### 2.3. Testing Procedure

**Figure 2:**
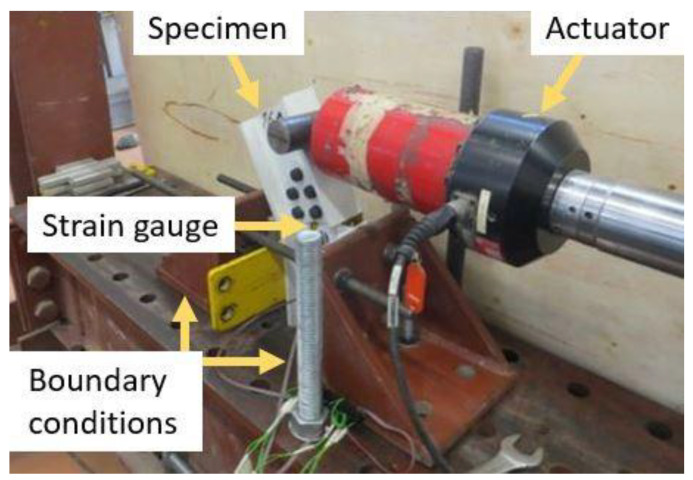
Test setup configuration system.

One of the two halves of each specimen was completely fixed to an external restraining structure, whereas the other half was free to move and was only restrained by the studied joint. Force was applied horizontally at 50 mm vertically from the free edge of the non-constrained halve. A hydraulic actuator with a 100 kN force range was used. A steel cylinder coupled to the hydraulic actuator was used as a lineal loading tool. The test was displacement controlled and the load was indirectly applied through an imposed horizontal displacement at 1 mm/min. A rosette strain gage (3 strain gages of 120 Ω at 45° and 90° connected with 3 wires and temperature-compensated for glass fiber composites) was placed on the central point of the joint (web connector on one side of the connection) to determine the main strain values and their directions. Figure 2 shows the test setup configuration in detail.

## 3. Finite Element Modelling

General purpose commercial simulation software (ABAQUS^®^ 2020, Dassault Systems, Pawtucket, RI, USA) was used to implement a numerical model that is able to be translated to many other simulation tools. In particular, Abaqus/Standard analysis was used in all models in order to assure this simplicity.

Regarding the geometric definition of the model, all parts were simulated using 3D parts. Screws were simplified as cylindrical parts, and nuts and washers were not considered in the simulation. Experimental evidences promoted modelling GFRP pultruded profiles with an orthotropic material. Linear-elastic behavior and transverse isotropy were assumed. Table 6 gathers the nine engineering constants used to model pultruded GFRP material. The handmade carbon fiber laminate was simulated as a linearly-elastic isotropic homogeneous and the considered properties to be introduced in the numerical model are summarized in Table 7. Finally, the steel bolts’ material was elastic-perfect plastic as defined in Table 7.

**Figure 3:**
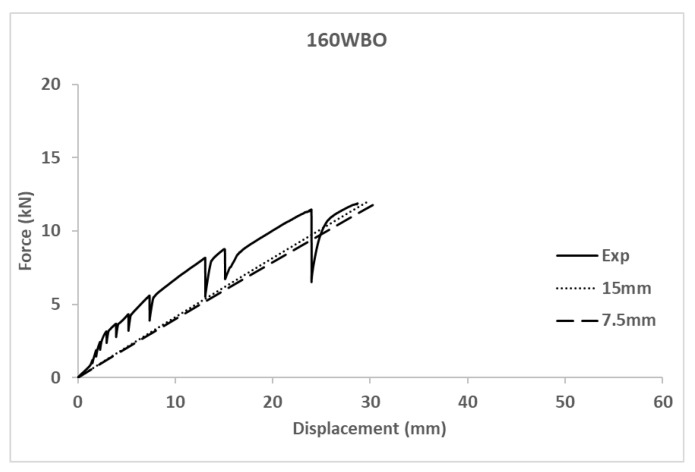
Mesh-convergence analysis on numerical result.

A mesh convergence analysis was performed through analyzing a specific case (160WBO) with two different mesh sizes: 15 mm and 7.5 mm. Compared results in terms of force-displacement curves are shown in Figure 3. Maximum stress values had a variation below 10%. In conclusion, the mesh size of 15 mm was accepted as a balanced option between simulation accuracy and computational cost, which was also limited to a maximum calculation time of 8 h on in an Intel^®^Core^TM^ i7-7500 CPU @ 3.8 GHz with 16 GB RAM memory running Windows10.

GFRP profiles and CFRP connection plates were meshed as 3D solid elements (C3D10, 10-nodes quadratic tetrahedron). Bolts and connection plates were meshed with 3D solid elements (C3D8R, 8-nodes linear brick integration with hourglass control). A total number between 600 and 19,300 elements were used depending on the joint type.

Contacts between parts were geometrically defined to assure that all parts were in contact to the adjacent ones. The contact between the GFRP profiles, the connection plates and the bolts were defined as surface-to-surface contacts with friction model interaction. These contacts considered a Coulomb friction model with a friction coefficient value of 0.2 [51,52]. On the other hand, a tie constraint, was defined to model adhesively joints. This last definition completely reflects experimental observations up to brittle failure.

Regarding boundary conditions, real displacement restraints of the laboratory conditions (see Figure 2) were recreated in the numerical model by completely fixing the external surfaces of the flanges of the restrained halve. The load was applied as an imposed displacement in global horizontal direction. Static analysis was carried out.

The maximum number of times increments for the analysis was set to 200, with an initial arc length increment of 0.01. The solver employed a direct equation solver and the full Newton technique.

As a result, the implemented FE model provided a simplified tool capable of reproducing complex experimental tests in such a way that future connections between GFRP profiles could be studied numerically, thereby saving the cost of experimental campaigns. In comparison with other existing models, it is a simplified approach aimed to demonstrate the capabilities of this easier simulations for representing global response of GFRP pultruded profiles joints. However, there are more accurate models for specific studies, like the one presented by Fascetti et al. [15], which dealt with the flange-web shear response of pultruded profiles, the one presented by Li et al. [43], which dealt with stress distribution in in-plane bolted connections through a 2D model or the model by Yu et al. [44], which used Abaqus/Explicit to represent dynamic response of composite single-bolted joints.

**Table 6:** The mechanical properties of the GFRP profile. Values obtained to fit the numerical model.

*E*_1_ (GPa)	*E*_2_ = *E*_3_ (GPa)	*v*_12_ = *v*_13_	*v*_23_	*G*_12_ = *G*_13_ = *G*_23_ (GPa)
11.2	1.6	0.27	0.33	0.6

**Table 7:** Mechanical properties of steel [53] and CFRP (values obtained to fit numerical model).

Material	Characteristics	Value
Steel	Young’s Modulus (GPa)	210
	Poisson’s rate	0.29
	Yield stress (MPa)	1100
	Plastic strain	0
CFRP	Young’s Modulus (GPa)	227
	Poisson’s rate	0.26

## 4. Results

### 4.1. Experimental Results

**Figure 4:**
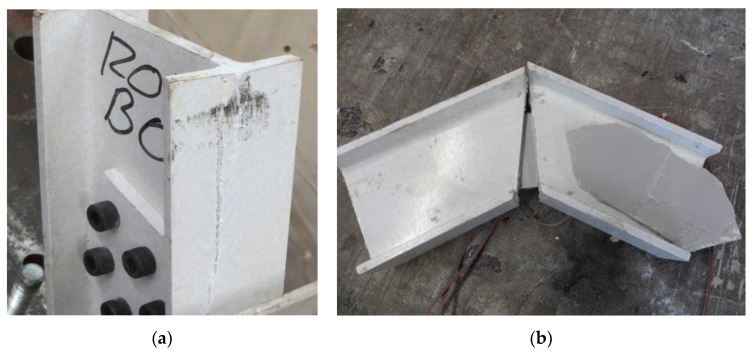
Failure modes. (**a**) Local failure; (**b**) Debonding failure.

Table 8 summarizes the main experimental results including maximum load-bearing capacity (F_max_), horizontal displacement of the load application point at the maximum force (d_max_), maximum and minimum principal strain values in the central point of the web connector at the maximum force (ε_1_ and ε_2_) and orientation of this strain vector with respect to the longitudinal symmetry axe of the connection plate (θ_1_). Finally, the failure mode is also included in Table 8. All bolted specimens failed because of local web-to-flange shear failure (see Figure 4b) whereas all bonded specimens failed because of debonding of the web connection plate (see Figure 4a).

The first qualitative experimental result was that the load-bearing capacity of adhesively connected joints was far lower than bolted ones around five times for the tested specimens. The second observation showed there was no clear influence of adding the flange connection to the web one in terms of load-bearing capacity, although flanges-connected specimens were stiffer.

**Figure 5:**
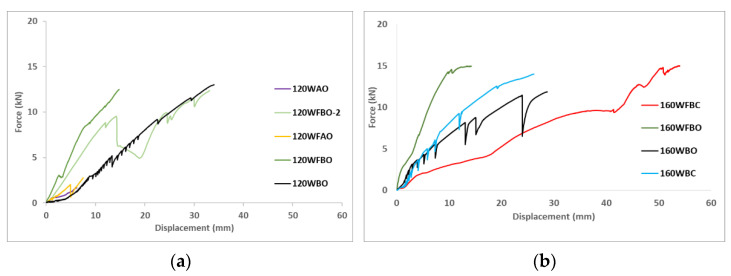
Force-displacement plots (**a**): specimens with the angle of 120° (**b**): specimen with the angle of 160°.

Figure 5 represents the force-displacement plots of all specimens. It can be seen that all the graphs had an uptrend. Some cases (120WBO, 160WBC and 160WBO) showed continuous saw-like curve associated with the progressive settling of bolted connection with imperfect holes that allowed certain punctual sliding that was traduced into a force decrease that was automatically restored to the previous force because the rest of the bolts bore the released force. This phenomenon is supported by the fact that no external additional displacements were recorded during these responses.

**Table 8:** Experimental results.

Specimen	F_max_ (kN)	d_max_ (mm)	ε_1_ (×10^−6^)	ε_2_ (×10^−6^)	θ_1_ (°)	Failure Mode
**120WAO**	1.53	10.36	152	−237	77.6	Debonding failure
**120WBO**	13.01	34.56	1610	−1515.8	7.7	Local failure
**120WFAO**	2.76	13.22	10.3	−1	97	Debonding failure
**120WFBO**	12.36	16.42	577.2	−185.3	4.1	Local failure
**120WFBO-2**	12.48	20.25 *	394	36	9.7	Local failure
**160WBC**	14	43.63	1660	−1945	36.4	Local failure
**160WBO**	11.88	28.77	1533	−1051	40.1	Local failure
**160WFBC**	15.01	55.52	182	−578.9	82.32	Local failure
**160WFBO**	14.96	35.52	1711	−1719	38.8	Local failure

* This value was corrected because of the actuation tool slides from a bolt cap to an inter-bolt flange position, causing a sudden increase of the displacement with a transitorialunload-reaload process according with Figure 5.

The maximum load-bearing capacity was reached by the 160WFBC specimen (15.01 kN) and the 120WAO specimen reached the lowest ultimate load (1.53 kN).

**Figure 6:**
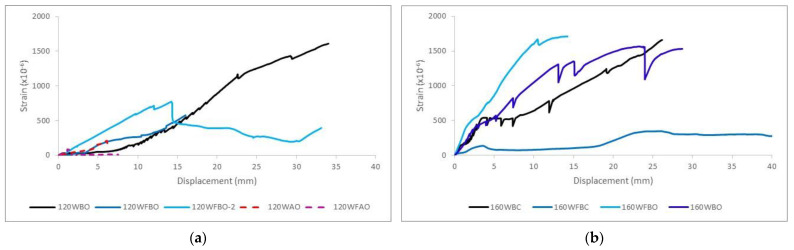
Strain-displacement plots (**a**): specimens with the angle of 120° (**b**): specimens with the angle of 160°.

Figure 6 shows the first principal strain-displacement plots for all specimens. Observing the plots, it is clear that the connection plate played its role assuming and transmitting increasing stresses between the two halves of the connection during mechanical testing.

### 4.2. Numerical Results

**Figure 7:**
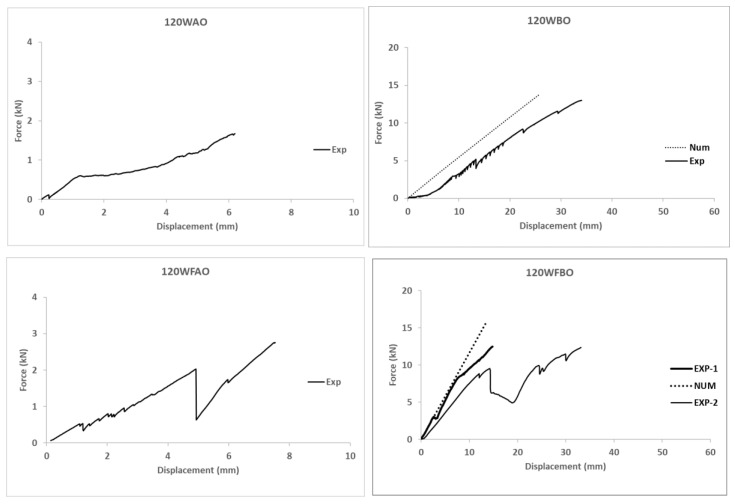

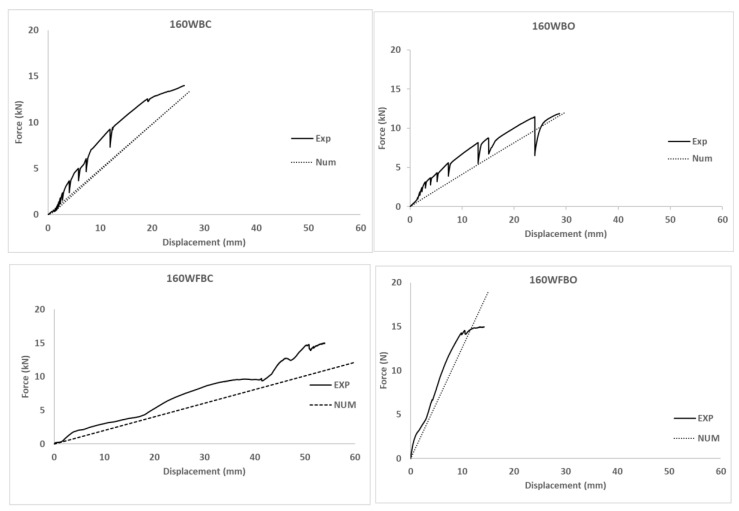
Force-displacement plots for all experimental and numerical models.

Figure 7 shows the force-displacement curves for all numerical models, compared with experimental results. It can be seen that there is agreement between the numerical model and the experimental results in terms of stiffness, with an average difference between both studies below 10%. It shows the numerical model correctly captured the mechanical response of the connection in the elastic phase before failure. Numerical curves for adhesive cases are not compared in Figure 7 because of the unexpected experimental brittle failure of these connections, although results are used to comment about the possible reasons of the observe response.

**Figure 8:**
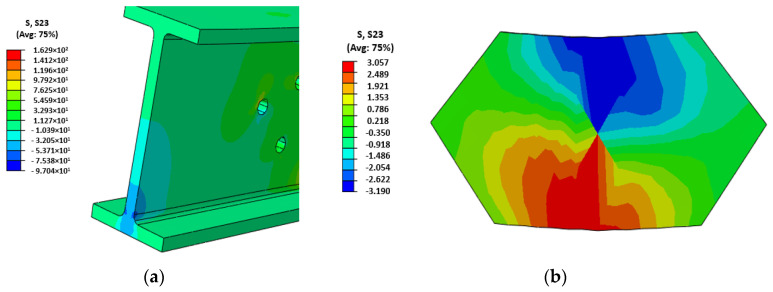
Shear stress (MPa) plots (**a**) Local web-to-flange shear failure for case 160WBC; (**b**) Shear stress distribution in the contact surface of the web connection plate of specimen 120WAO.

The maximum load-bearing capacity was not specifically calculated but it may be justified on the basis of the local web-to-flange failure of profiles that was experimentally observed and numerically (see Figure 8a) represented by a shear stress (S23, in the web plane) over the range of 30 MPa–50 MPa, which were the maximum shear strengths reported by Neagoe [46] depending on the testing standard. It is assumed the failure happened when a continuous area crossing the web thickness overpassed the previously mentioned values. These values were obtained for the same profiles used in the current research.

None of the profiles reached their tensile strength, nor did the bolts reach their shear strength in any of the simulations, which showed a continuous stress distribution throughout all simulation. In-plane shear stresses in the connection plate bonded face is presented in Figure 8b.

## 5. Discussion 

Considering the experimental results (see Figure 5 and Table 8) obtained from the tests and the corresponding failure modes in Figure 4, together with the numerical results in Figure 7 and Figure 8, it is clear that different parameters influenced the behavior of GFRP connections. The analysis of this influence is presented in this section.

First of all, the repeatability of the tests is analyzed by comparing 120WFBO and 120WFBO-2 specimens. Both reached almost the same maximum load (see Table 8) and showed similar apparent stiffness (around 1 kN/mm). Even the point at which the slope of the force-displacement curve changed was located at the same load level (around 9 kN). Thus, repeatability of the testing procedure can be assured although there is variation in the displacement measurements due to the handy execution of the bolted connections that allowed slight sliding of the bolts inside the hole during the initial settling process. After that point, stiffness values may be calculated with confidence although total displacement at maximum load (d_max_ in Table 8) may not be directly comparable without fitting this previously described initial settling, or the actuation tool slide described in Table 8.

(a) Influence of the geometry (angle between profiles). Comparing the equivalent specimens with different angles (120WFBO vs. 160WFBO and 120WBO vs. 160WBO) it was observed that 160WFBO with 3.6 kN/mm was stiffer than 120WFBO with 1.2 kN/mm, whereas no significant difference was observed between 120WBO and 160WBO, which showed an equivalent stiffness value around 0.5 kN/mm. This fact seems to indicate that the presence of the flange connector caused the change in the mechanical response when modifying the angle between profiles. It can be explained because of the greater performance of the straighter carbon FRP flange connector of 160WFBO specimen, with a resulting apparent stiffness of 3.6 kN/mm (3.1 kN/mm increase respect to the case of 160WBO), in comparison with the flange connector of 120WFBO specimen, that showed less stiffness increase (0.7 kN/mm respect to 120WBO case) because of the bending of the carbon fibers during the production phase of the later one. In conclusion, this experimental evidence indicated that flange connectors were more effective for larger angles between profiles. It is possible to increase the connection stiffness improvement up to 4 times for the greatest tested angles between profiles (160°) respect to the minimum tested angle (120°).

(b) Influence of the connection type (bolted vs. adhesive). According to the experimental results and for the considered combinations of materials it is clear that bolted connections achieved greater load bearing capacity than adhesive ones. However, the pure debonding failure observed in the adhesive connections indicated a poor execution performance which makes these results not comparable to the ones existing in the literature (see [26]) in terms of load-bearing capacity although the fragile debonding failure is really characteristic of this type of joints. Before failure, comparable specimens with web only connections (120WAO vs. 120WBO) had really similar responses in terms of force-displacement curve, so the connection type showed no influence. Specimens with bolted flange connection (120WFBO) had a stiffer response (1.2 kN/mm) than the analogous tests on samples with adhesive connection (120WFAO with an apparent stiffness of 0.3 kN/mm) from the very beginning of the test, indicating that the debonding of the flange started from the beginning of the experiment because the out-of-plane debonding strength was lower than the corresponding in-plane shear strength of the used epoxy resin (see [50]). This fact supports the idea of improving the surface treatment before executing adhesive connections in future researches. In the same line, there was no difference between including or not including flange connection for adhesive specimens (120WAO vs. 120WFAO) because of the progressive out-of-plane debonding process of the flange, which did not contribute to the mechanical response. All together, these facts seem to indicate that the adhesive installation of the flange connection did not work for the described experimental campaign. In addition, adhesive connections were always associated with fragile debonding failure types (see Table 8 and Figure 3), in accordance with existing literature (see [25,26]), whereas bolted connections reached the local failure of the GFRP profile at the web-flange edge as reported by other researchers (see [8,14,15]) who studied this phenomena concluding that the roving-rich area in the web-flange junction was the base cause of this response.

(c) Influence of the testing orientation (opening or closing the angle). According to the experimental results of the comparable specimens (160WFBC vs. 160WFBO and 160WBO vs. 160WBC) no clear influence was observed for web only connected specimens. This evidence showed a symmetric response of web-connected cases in front of bending sign change. In contrast, a clearly stiffer response for opening testing orientation was observed when both flange and web were connected (see point a in this section). These results have to be analyzed carefully although strain measurements (see Table 8) seem to prove that the web supported lower stresses when flange was included, pointing out the usefulness of the flange connectors in bearing joint efforts. Nevertheless, both testing orientations reached really similar load-bearing capacities, of 15 kN. This fact, supported the idea that the local failure of web-flange area of the GFRP profile defined the end of the test instead of the joint itself. In addition, it is thought that the configuration that tended to open the angle made the flange connection to better collaborate, removing mechanical requirements from the web connection and reducing the corresponding measured strains. On the opposite, testing with the orientation that tended to close the angle of the joint caused that the initially bended fibers were more likely to broke because of local bending effects, making the flange connector to contribute less respect to the case of opening the initial angle between connected profiles. This evidence is also related to the previous observation that straighter flange connectors behaved stiffer than others with closer angles. This justifies the conclusion that the mechanical stiffness of flange connectors increased when the loading configuration did not increase the initially existing curvature of the fibers, that may cause local bending failure. However, further tests to confirm this point are required in future campaigns to confirm this provisional conclusion, which has no literature evidences to compare with as long as connection angle is not a commonly tested parameter.

(d) Influence of the connectors (web only connector vs. web and flange connectors). First of all, it has to be noticed that the adhesive connection was not efficient enough to be taken into consideration according to the previous discussion, so it is discarded from the current analysis. Thus, comparing analogous specimens with bolted connections (120WFBO [-2] vs. 120WBO, 160WFBC vs. 160WBC and 160WFBO vs. 160WBO) it is clear that including the flange connector motivated an initial stiffer response of the joint when the test configuration tended to open the angle between the profiles, so this flange connector collaborates from the very beginning to restrain deformation as previously suggested. The load bearing capacity was not really affected as long as the failure mode was associated with local flange-to-web shear damage. For the specimens tested in the opposite orientation, this effect was not observed because of the possible failure of flange fibers in local bending, as supported before. However, 160WFBC was the most deformable joint among the tested ones. This fact can be justified because of an incomplete fixation of the bottom part of the joint to the external testing frame during the test. This possibility would also explain the lower strains measured throughout the test. Nevertheless, it has to be reminded at this point, that total displacement measurements were less confident than force and strain measurements.

From a practical point of view, the production complexity and the cost of the flange’s connectors together with the observed limited performance of this solution advised against using them in real applications.

(e) In relation to the orientation and magnitude of the first principal strain at failure time, the specimens which had an angle of 160° between GFRP parts had a first principal strain between 1500 × 10^−6^ and 1700 × 10^−6^ with an angle in the range between 35°and 40° respect to the longitudinal symmetry axe of the web connection plate, with the exception of 160WFBC specimen. This case showed an anomalous global mechanical response (far more flexible than expected, as commented before) which was translated into lower strain measurements that indicate that the web connection plates were not subjected to the same stress level as other specimens with the same angles between profiles.

For little stress levels, the orientations calculated from strain measurements were highly affected by the relative variability of measurements. A little variation of 10 × 10^−6^ strain measurement caused variations of the angle of the principal strain over 90°. Thus, adhesively connections 120WAO and 120WFAO, which showed low strains corresponding to the little load-bearing capacity recorded, should not be analyzed in terms of strain value or orientation.

Moving to the specimens with the angle of 120°, 120WBO, 120WFBO and 120WFBO-2 had a similar orientation of first principal strain (4–10°), but showing far lower values of strain (between 400 × 10^−6^ and 600 × 10^−6^) for the cases with flange connection, indicating that a significant part of the applied efforts was supported by the flange reducing the stresses, so the strains, in the web connectors, which reached up to 1600×10^−6^ in the case 120WBO but only 110×10^−6^ for 120WFBO average cases (the strain was reduced up to 93%). Strains were reduced almost completely to for 120WAO case and a reduction of 70% was recorded for 160WBC case when adding flange connector. This value is in the range of the previously reported for 160WBO and 160WBC. This phenomena of redistributing efforts between flanges and web connectors are well stated in the literature and completely comparable to the steel structures joints in the elastic phase. Thus, this evidence supported the idea of a proper response of the bolted joints up to the local failure of the flange-to-web junction.

Analyzing the influence of additional parameters, it is concluded that some variables apart from the analyzed ones may affect the presented results, like the thickness of CFRP, the free gaps in holes for bolted connections or the (non)preparation of the surface of the profile for adhesive joints. All these parameters should be assessed in future researches.

Finishing with the discussion of the experimental results, it has to be highlighted that the same failure mode observed for bolted joints in the current research, which corresponded to the local failure of the web-to-flange junction, was also reported by other researchers like Fascetti et al. [15] or Turvey et al. [14]. In addition, the second one studied I-shape pultruded glass fiber profiles slightly bigger (203 × 203 × 9.5 mm) than the ones used in the current research, reaching load-bearing capacities of the same order of magnitude (around 20 kN). These evidences validated the observations and analysis performed on the bases of the failure mode.

Hence, adding a flange connector helps to uniform the manually executed connection, providing an experimental response closer to the theoretically expected one, so being easier to reproduce by numerical models.

Regarding the numerical simulations, the model predicts the mechanical response of the specimens that had flange connectors in a more accurate way than the ones with only web connectors. In fact, the average square of the distance between experimental and numerical curves in Figure 8 are, in kN^2^, 11.53, 11.37, 4.04, 2.35, 4.1 and 3.06 for 120 WBO, 120WFBO, 160WBC, 160WBO, 160WFBC and 160WFBO respectively. It was calculated by adding the square value of the force difference between the curves in the simulation output displacement calculated points. It is shown that connections with 160° had better fitting. The average relative error associated to these differences is in the range between 10% and 20% if the full curve is compared. In addition, it has to be noted that the results of the numerical model confirmed that bolts, connection plates, and GFRP profiles reached stress levels far below their strength, except for the weaker part of the GFRP profiles, which is the web-flange connection as previously reported by other researchers [14,15,46].

Finally, the analysis of the simulations of the adhesive connections showed that a shear connection strength of 3.6 MPa (see Figure 7b) was reached, whereas the provider of the adhesive reported a shear strength between steel plates above 14 MPa (see [54]). Thus, it is concluded that the surface of the GFRP profiles was not properly prepared before the installation of the adhesive connection parts. In fact, the reported shear strength in the current campaign is also far below the strengths reported in other studies (see [25,26]).

## 6. Conclusions

Nine GFRP joint specimens with different configurations (bolted joints, adhesive joints, web joints, web and flange joints, and two different angles between profiles) were experimentally tested and numerically simulated. The following conclusions were obtained:

- In general, flange connectors are more effective for larger angles between profiles. These are also more effective when the internal bending moment tends to widen the angle between profiles. It indicates that the mechanical stiffness of flange connectors increased when the loading configuration did not increase the initially existing curvature of the fibers.
- Flange connection increases joint stiffness by 7.6 times but only shows a slight improvement in load-bearing capacity, around 26%, when the failure is controlled by a local profile collapse.
- The inclusion of a flange connection redistributes stresses in the joint, promotes a more uniform joint response, and unloads the web connector as measured by strain gages. A significant part of the applied efforts is supported by the flange, reducing the stresses in the web. Flanges reduced the strain in web over 70% respect to the cases 120WAO, 120WBO and 160WBC.
- Adhesive connections are always associated with fragile debonding failure types.
- Numerical simulation accurately predicts the mechanical response in terms of force-displacement behavior, showing an average relative error between 10% and 20% when assessing the full testing curves. However, the current model is not able to capture the local web-to-flange shear failure in a direct way, but it may be assessed by comparing the corresponding results with the material strength values.
- According to the mesh-convergence analysis, it is clear that there is no mesh influence on the findings.

Further research may include defining the complex web-to-flange shear failure in the numerical model through using a more detailed material definition or including an additional material in the web-to-flange connection area that represents this weaker part of pultruded profiles by replacing the originally defined material. Setting the mechanical properties of this representative part opens a significant future research line with the aim of properly simulating composite pultruded profiles.

## Figures and Tables

**Table 1:** Details of the specimens.

Specimen	Angle (°)	Connection Position	Connection Type	Force Direction
**120WAO**	120	web	Adhesive	open
**120WBO**	120	web	Bolt	open
**120WFAO**	120	web & flange	Adhesive	open
**120WFBO**	120	web & flange	Bolt	open
**120WFBO-2**	120	web & flange	Bolt	open
**160WBC**	160	web	Bolt	close
**160WBO**	160	web	Bolt	open
**160WFBC**	160	web & flange	Bolt	close
**160WFBO**	160	web & flange	Bolt	open

**Table 2:** The mechanical properties of the GFRP profile. Data from [31].

Property	Value	Units	Testing Method
**Flexural**			
Ultimate strain	2.10 ± 0.05	%	EN ISO 14125:1998
Strength	734 ± 39	MPa	
Modulus of elasticity	35.0 ± 2.1	GPa	
**Tensile**			
Ultimate strain	1.37 ± 0.11	%	EN ISO 527-1:2012 EN ISO 527-4:1997
Strength	520 ± 27	MPa	
Poisson’s ratio	0.27 ± 0.02		
Modulus of elasticity	38.0 ± 1.4	GPa	
Effective shear modulus	3.98 ± 0.26	GPa	

**Table 3:** Properties of the carbon fiber used to produce CFRP laminates for flange connection [47].

Property	Value
Density (kg/m^3^)	1.102
Creep resistance ^1^ (MPa)	14.5
Deformation stress ^1^ (%)	2
Elasticity module ^1^ (MPa)	717
Ultimate resistance ^1^ (MPa)	17.2
Poisson index ^1^	0.48

^1^ Based on testing of cured samples per ASTM D 638 at 20 °C (72 °F) and 40% relative humidity.

**Table 4:** Properties of the epoxy resin used to produce CFRP laminates for flange connection [48].

Property	Test Method	Value
Elongation (%)	ISO 527-3	3
Tensile Strength (MPa)	ISO 527-3	27
Tensile Modulus (MPa)	ISO 527-3	1350
Compressive Strength (MPa)	ISO 604	65

**Table 5:** Properties of the epoxy resin used in adhesive connections [50].

Characteristics	Test Method	Value
Density (g/cm^3^)	-	1.4
Compressive resistance (N/mm^2^)	UNE-EN 12190	73
Young modulus (Compression) (N/mm^2^)	UNE-EN 13412	8700
Young modulus (Flexure) (N/mm^2^)	UNE-EN ISO 178	4260
Linear shrinkage (%)	UNE-EN 12617-1	0.03

## Data Availability

Not applicable.
